# Cytokine Release Syndrome and Associated Acute Toxicities in Pediatric Patients Undergoing Immune Effector Cell Therapy or Hematopoietic Cell Transplantation

**DOI:** 10.3389/fonc.2022.841117

**Published:** 2022-03-24

**Authors:** Susanne H. C. Baumeister, Gopi S. Mohan, Alaa Elhaddad, Leslie Lehmann

**Affiliations:** ^1^ Boston Children’s Hospital, Division of Pediatric Hematology-Oncology, Boston, MA, United States; ^2^ Department of Pediatric Oncology, Dana-Farber Cancer Institute, Boston, MA, United States; ^3^ Harvard Medical School, Boston, MA, United States; ^4^ Division of Pediatric Critical Care, Massachusetts General Hospital, Boston, MA, United States; ^5^ Children’s Cancer Hospital of Egypt, National Cancer Institute Cairo, Cairo, Egypt

**Keywords:** cytokine release syndrome (CRS), immune effector cell associated neurotoxicity syndrome (ICANS), immune effector cells (IEC), CAR T cells, blinatumomab, pediatric hematopoietic cell transplantation (HCT), haploidentical cell transplantation (Haplo-HCT)

## Abstract

Immune effector cells (IEC) are a powerful and increasingly targeted tool, particularly for the control and eradication of malignant diseases. However, the infusion, expansion, and persistence of autologous or allogeneic IEC or engagement of endogenous immune cells can be associated with significant systemic multi-organ toxicities. Here we review the signs and symptoms, grading and pathophysiology of immune-related toxicities arising in the context of pediatric immunotherapies and haploidentical T cell replete Hematopoietic Cell Transplantation (HCT). Principles of management are discussed with particular focus on the intersection of these toxicities with the requirement for pediatric critical care level support.

## Introduction

Hematopoietic Cell Transplantation (HCT) is the oldest and most fundamental example of cell-based immunotherapy. It has become a cornerstone for the treatment of high-risk hematologic malignancies, certain pediatric solid tumors, benign hematopoietic disorders, immunodeficiencies and defined metabolic disorders. Specific phenomena associated with the influx, rapid expansion and activity of immune effector cells transferred during HCT, such as immune reconstitution syndrome and engraftment syndrome, have been well described although they are relatively uncommon. With the advent of post-transplant cyclophosphamide (PTCy) as a method to utilize T-cell replete mismatched grafts, the use of haploidentical donors has increased exponentially, and cytokine release syndrome (CRS) is seen not infrequently. Outside of HCT, the use of T-cell engager monoclonal antibodies (mAb), chimeric-antigen receptor T-cells (CARTs) and other targeted cellular immunotherapies have also demonstrated potential for remarkable clinical efficacy and revolutionized therapeutic options, particularly for hematologic malignancies. The therapeutic power of such approaches has increased the incidence of significant inflammatory, immune-mediated toxicities including CRS, Immune Effector Cell-associated Neurotoxicity Syndrome (ICANS), and less commonly, a Hemophagocytic Lymphohistiocytosis (HLH)-like syndrome. While there have been remarkable advances in our ability to manage these toxicities, the field continues to evolve rapidly in terms of understanding both the pathophysiology common to many different immune effector cell therapies and the discrete toxicities associated with specific immune cell subsets, targets, genetic modifications, and manufacturing of the infused product. Importantly, features of immune activation arising from the recognition of targeted cells may have significant overlap with other clinical scenarios such as bacterial sepsis and evaluation and exclusion of alternative etiologies is critical and frequently challenging. Here we review the signs and symptoms, grading, pathophysiology, and principles of management of immune-related toxicities arising in the context of pediatric HCT, IEC and immune-cell engaging therapies, particularly as it relates to pediatric critical care.

## Signs, Symptoms and Grading of CRS and ICANS

The definition of CRS has evolved over time and has been iterated within the Common Terminology Criteria for Adverse Events (CTCAE). Initial trials involving CART cell therapies utilized a variety of toxicity grading systems for individual trials. These included standard CTCAE v 4.0 criteria (1), CTCAE v 5.0 criteria (2), Lee toxicity criteria (3), Penn grading scale (4, 5), MSKCC criteria (6) and CARTOX criteria (7). This unfortunately precluded a consistent and comparable assessment of CART cell-related toxicities across different products, trials, and institutions (8). In response to the need for a uniform grading system across clinical trials and to allow accurate capture of the real-world experience of commercial CART cell products, the American Society for Transplantation and Cell Therapy (ASTCT) convened an expert panel in 2018. This resulted in the ASTCT Consensus Grading for CRS and neurologic toxicity with immune effector cells (9). These grading criteria are now widely adopted in the field, although it should be noted that they pertain to grading and do not include management recommendations. The ASTCT criteria define CRS as a “supraphysiologic response following any immune therapy that results in the activation or engagement of endogenous or infused T cells and/or other immune effector cells. Symptoms can be progressive, must include fever at the onset and may include hypotension, capillary leak (hypoxia) and end organ dysfunction” (9).

As noted above, fever is necessary for the diagnosis of CRS and is defined as a temperature ≥ 38.0°C. Higher fevers, exceeding 40.0°C, can often be seen and be persistent for days. Persistent fevers in the context of CRS have generally been defined as lasting >3 days (10, 11). Fever may or may not be associated with constitutional symptoms such as rigors, fatigue, malaise, anorexia, myalgias, arthralgias, nausea, vomiting and headaches. End-organ dysfunction is variable, but frequently involves the cardiovascular system, manifesting as hypotension, widened pulse pressure, tachycardia, and increased cardiac output that can progress to cardiac failure. Respiratory involvement with tachypnea and hypoxemia requiring oxygen and ventilatory support is not uncommon. Additional organ dysfunction may be present including hepatic involvement (transaminitis and/or hyperbilirubinemia), renal dysfunction, gastrointestinal symptoms (nausea, vomiting and diarrhea), skin rashes and coagulopathy with elevated D-dimer, hypofibrinogenemia and the potential for bleeding (3, 12) (**Table 1**). The onset and resolution of CRS manifestations are in part dependent on the specific immune effector cell therapy administered or engaged, but generally occur early, within the first 1-2 weeks after product administration. However, late onset CRS has been described (13) and should be considered in the differential diagnosis of patients presenting within an 8-week window of infusion.

**Table 1:** Signs and symptoms of CRS and ICANS.

System	Symptoms
Constitutional	**Fever ≥38.0°C**, malaise, fatigue, myalgias, arthralgias
Cardiovascular	**Hypotension**, tachycardia, arrythmia
Pulmonary	**Hypoxia**, tachypnea
Hepatic	Transaminitis, hyperbilirubinemia
Renal	Azotemia, Acute renal insufficiency
Gastrointestinal	Nausea, anorexia, vomiting, diarrhea
Coagulation	Hypofibrinogenemia, D-dimer elevation, PT/PTT prolongation, bleeding
Skin	Rash
Neurologic	Headaches
	Mild: Somnolence, lethargy, disorientation to time and place, impaired attention or short-term memory, dysgraphia, tremor, mild expressive aphasia
	Severe: Global aphasia, coma, seizures, increased tone, focal weakness, cerebral edema/intracerebral hemorrhage

In bold are headers and cardinal symptoms of CRS.

Neurotoxicity or ICANS frequently occurs following CRS but is excluded from the definition of CRS. This is due to limited mechanistic data linking the two, the propensity of ICANS to occur after the peak of CRS and unresponsiveness to effective therapeutic interventions mitigating CRS such as Tocilizumab administration. However, the majority of patients who develop ICANS experienced preceding CRS and thus CRS is considered a possible initiating event or cofactor (14). Symptoms of ICANS can vary from mild to severe or even lethal, but are typically transient, particularly in pediatric patients. These may include headaches, word finding difficulties, dysphasia or frank aphasia, mental status changes ranging from confusion and hallucinations to somnolence or coma, tremor, dysmetria or motor weakness and possible seizures. Although generally reversible, rare fatal events involving cerebral edema and hernation have occurred (15) (**Table 1**).

**Figure 1:**
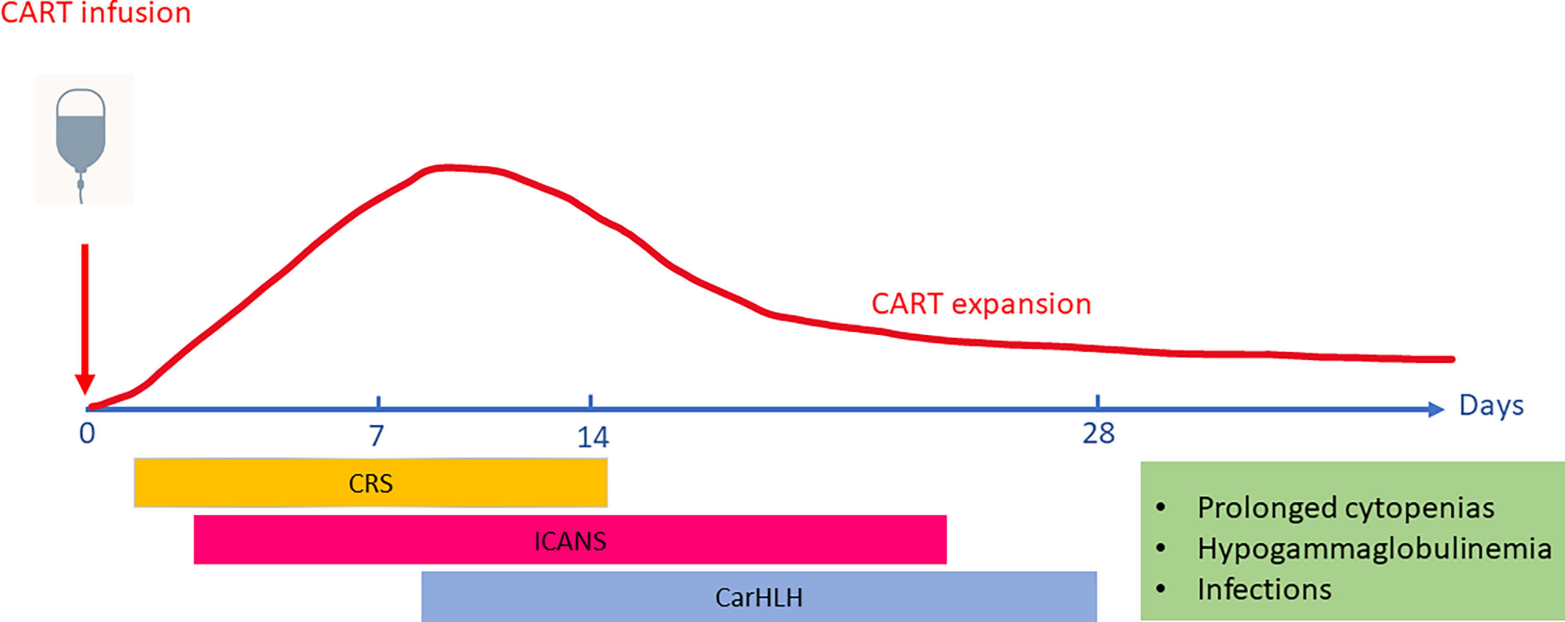
Timing of Toxicities associated with CART cell (Chimeric-Antigen Receptor T-cell) therapies. CAR T cells typically expand and peak in the peripheral blood 1-2 weeks after infusion. The onset and peak of Cytokine Release Syndrome (CRS) generally occurs in the first week after CART infusion and precedes the onset of ICANS (Immune Effector Cell-associated Neurotoxicity Syndrome). While CAR-associated Hemophagocytic Lymphohistiocytosis-like syndrome (CarHLH) manifestations may overlap with CRS, it has also been described as a separate entity emerging after resolution of CRS. Upon resolution of acute CART toxicities, patients are at risk for prolonged cytopenias, hypogammaglobulinemia and infectious risks due to ongoing B-cell aplasia mediated by persistence of functional CART cells.

The most extensive experience with CRS and ICANS in pediatric patients is from the CD19-targeted autologous CART cell product Tisagenlecleucel. In the reported safety outcomes for pediatric acute lymphoblastic leukemia (ALL), 55% of patients experienced CRS (≥ Grade 3 in 16%) in the real-world setting as reported to the Center for International Blood and Marrow Transplant Research (CIBMTR) (n=255) (13), compared to 77% (≥ Grade 3 in 48%) on the pivotal ELIANA trial (16). Median time to onset was 6 days (range 1-27) in the CIBMTR cohort and 3 days (range 1-22) in the ELIANA trial respectively. Median duration of CRS was reported as 7 days in the CIBMTR cohort (range 1-76 days) and 8 days (range 1-36) in the ELIANA trial. Neurotoxicity was observed in 27% of patients (≥ Grade 3 in 9%) (CIBMTR) and 38% (≥ Grade 3 in 12.7%) (ELIANA) with a median time to onset of 7 days (range 1-80) (CIBMTR) and 8 days (range 2-489) (ELIANA) with a median duration of 7 days in both (range 1-94 in CIBMTR cohort) (13) (**Figure 1**).

In a pediatric trial of CD19-targeted CART cells with a CD28-costimulatory domain, any grade of CRS occurred in 80% and severe CRS (by Lee criteria (3)) in 16% of patients. Seventy-two % of patients experienced any neurotoxicity, with Grade 3/4 severe neurotoxicity in 28% (17). In a large study (n = 58) of children and young adults receiving CD22-targeted/4-1BB CART cell therapy for relapsed/refractory CD22+ B-ALL (18), 86% developed any grade CRS [≥ Grade 3 in 10% per Lee (3) and 24% by ASTCT grading criteria (9)]. Transient neurotoxicity occurred in 32.8% of patients with minimal severe neurotoxicity in 1.7% of patients.

Risk factors for the development of CRS include high disease burden, factors associated with robust early CAR T cell expansion and high CAR T cell dose (16, 19) (**Table 2**).

Risk factors for the development of ICANS include high disease burden, peak CART cell expansion, high grade CRS, high CART cell dose, extramedullary disease/CNS involvement, preexisting neurologic comorbidities, younger age, B-ALL, early fever onset and high concentrations of inflammatory cytokines within the first 3 days after CAR-T cell infusion (16, 20, 21) (**Table 2**).

**Table 2:** Risk factors associated with toxicity.

Toxicity	Risk factors
CRS	High disease burden
	Factors associated with robust early CAR T cell expansion (CD28 costimulatory domains)
	Higher CAR T cell dose
ICANS	High disease burden
	High grade CRS
	Peak CART cell expansion
	Extramedullary disease/CNS involvement
	B-ALL
	Younger patient age
	High CART cell dose
	Preexisting neurologic comorbidity
	Early fever onset
	High concentrations of inflammatory cytokines within 3 days of CART infusion

## Signs and Symptoms of CAR-HLH

More recently, CAR-associated HLH/MAS (CarHLH) has emerged as a separate entity which may overlap with CRS or occur as a late toxicity after resolution of CRS. In the context of CART therapy, HLH has been defined as peak ferritin >100,000μg/L (>100,000ng/mL) with at least two of the following criteria: a) Hepatic aminotransferases or bilirubin CTCAE grade ≥3, b) Creatinine CTCAE grade ≥3, c) Pulmonary edema CTCAE grade ≥3 and d) evidence of hemophagocytosis on bone marrow aspirate/biopsy (18, 22). HLH/MAS-like toxicities have occurred only in patients who had a history of CRS. This was initially described in the context of CD22-CAR T cells (particularly with CD4/CD8 T cell selection of the T cell product) for pediatric B-ALL where 32.8% of patients developed HLH-like manifestations prompting the use of anakinra in a subset of patients (18). CarHLH has also been observed in pediatric patients receiving CD19-CART cell therapy (23) as well as adults with B-ALL and diffuse large B cell lymphoma (24–26). The occurrence of serious bacterial infections with associated heightened inflammatory states may predispose patients to this late complication (27) (**Table 7**).

## CRS in Different Clinical Settings

### CRS in Patients Receiving Immune Effector Cells – Pathophysiology and Role of Biomarkers

**Figure 2:**
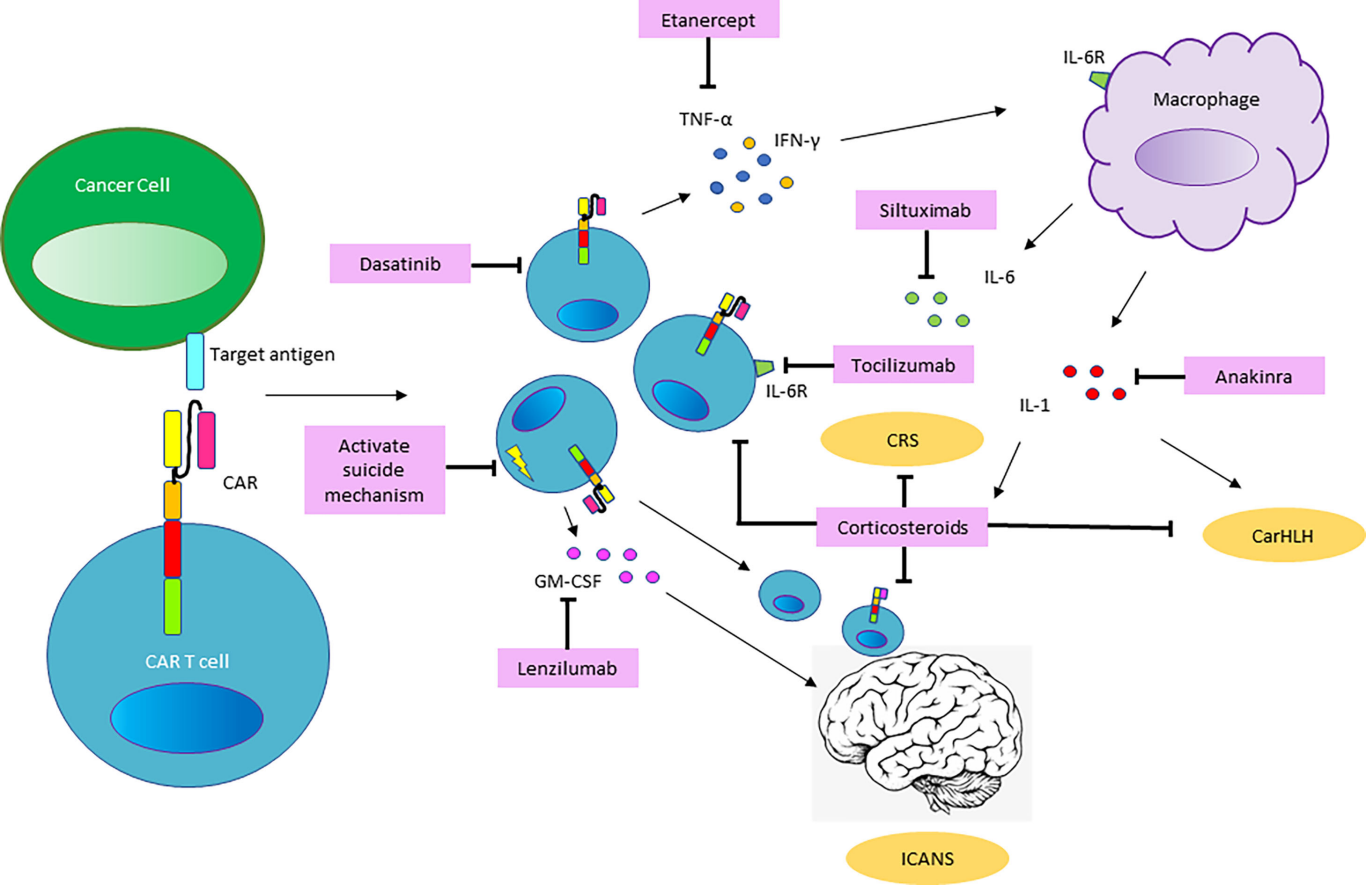
Mechanisms of Toxicity and Pathways for Intervention. Based on our current understanding of the pathophysiology involved in CART cell mediated toxicities, CART cells proliferate and produce inflammatory cytokines such as granulocyte-macrophage colony-stimulating factor (GM-CSF), interferon-γ (IFNγ), tumor necrosis factor-α (TNFα) and other soluble inflammatory mediators, upon recognition of the CAR-target antigen. This results in monocyte recruitment and activation of macrophages, which serve as the major source of Interleukin-6 (IL6) and Interleukin-1 (IL1), driving the systemic pathology of CRS. Penetration of cytokines and migration of CART cell and endogenous T cells into the CNS promote the development of ICANS. Shown in pink boxes are therapeutic tools for intervention in the pathways involved in CART-related toxicities. Blockade of the IL6-receptor (IL6R) by the monoclonal antibody (mAb) targeting the IL6R, Tocilizumab and the use of corticosteroids are well established in the management of CRS. Siltuximab, a mAb targeting IL6 may serve as an alternative agent to intervene in the IL6 pathway. The IL1-receptor (IL1R) antagonist Anakinra is emerging as a tool to preempt and treat CRS and has been utilized to treat CarHLH. The use of the TNF-antagonist Etanercept and GM-CSF antagonist Lenzilumab is not clinically established but may represent a therapeutic option based on the pathways involved. Preclinical data suggests that the kinase inhibitor Dasatinib may have a role in inhibiting CART cells and CART-mediated cytokine production. In CART cell constructs equipped with a suicide mechanism, pharmacologic activation of the suicide switch or mAb-based targeting of surface proteins included in the CART construct may be utilized to abrogate CART cell function in the setting of severe toxicity.

The pathophysiology underlying CRS in the context of immune effector cell therapies occurs in several phases and has been described as a continuum of CRS and ICANS (14). It has been most extensively reported for CART cell therapy and the manifestations are impacted by composition/costimulatory components of the chimeric receptor, the type of immune cell used as well as tumor type, antigen, and tumor burden. Following infusion, the initial phase is characterized by CART cell trafficking to the site of tumor cells bearing the antigen targeted by the CART cell *via* the chimeric receptor. Antigen recognition and CART cell activation then induce CART cell proliferation, cytokine production and activation of other cellular components in the tumor microenvironment. CART cell production of TNFα, IFN-γ and GM-CSF activates cells of the myeloid compartment which in turn secrete IL1, IL6, and inducible nitric oxide synthase (iNOS). Key cytokines such as IL-6 can also be produced by activated T cells, but it is now clear that cells of the macrophage and monocyte lineage are the major source of IL-6 and IL-1 in CRS (28, 29) (**Figure 2**).

CART-cell-mediated killing of target-bearing tumor cells and activation of endogenous immune cells leads to the systemic inflammatory response manifested as CRS with CART cell expansion and elevated cytokine levels in the peripheral blood. The migration of CART cells and endogenous T cells (30) into the CNS as well as penetration by systemic cytokines into the CNS results in ICANS in some but not all patients. Upon eradication of tumor cells and activation-induced cell death of T-cells, a decrease in serum cytokine levels and gradual reduction in the systemic inflammatory response marks the resolution of CRS/ICANS. Ideally ongoing tumor surveillance occurs through long-term memory CART cells.

Recent studies have focused on elucidating biomarkers that may predict severity of CRS prior to the development of critical illness as well as facilitate early distinction between CRS and sepsis. Disease burden at the time of CART-cell infusion is a known factor predicting severe CRS (31, 32). Evaluation of biomarkers in a cohort of 51 patients with ALL (n=39 pediatric) revealed elevated baseline Ferritin (median 1580ng/mL, range, 232-14,674) and CRP (median 1.2mg/dL, range, 0.12-29.4) in most patients, likely as a consequence of systemic inflammation and/or iron overload. However, the peak elevations of these markers were significantly higher in patients with Grade 4 or 5 CRS (Ferritin median 130,000ng/mL, range, 11,200-299,000 and CRP median 22.9mg/dL, range 16.0-37.1) than in those with Grade 0-3 CRS (Ferritin median 8,290ng/mL; range, 280-411,936 and CRP 16.2; range, 0.7-56.5). Grade 4 CRS was also strongly associated with hypofibrinogenemia (<150mg/dL). Furthermore, a peak in cytokine levels on a 24-cytokine profile including, IFNγ, IL6, IL8, sIL2Rα, sgp130, sIL6R, MCP1, MIP1α, MIP1β and GM-CSF during the first month after CART cell infusion was associated with Grade 4-5 CRS compared with Grade 0-3 CRS. The authors attempted to identify a cytokine constellation within the first 3 days after infusion that could predict the development of high-grade CRS. With IFNγ and sgp130 rising early in the course of CRS, decision tree models involving a combination of a) IFNγ and MIP1α, b) sgp130, MCP1 and Eotaxin c) IL10 in conjunction with disease burden were able to predict severe CRS before patients became critically ill (33).

One of the clinical challenges in the management of patients receiving IECs, HCT or T-cell engaging antibody therapies is the distinction of CRS from sepsis as the underlying pathophysiology. Most patients will have risk factors for sepsis including recent chemotherapy with associated neutropenia, presence of central venous lines and disease burden. Although the clinical presentations overlap, the management of these two entities is different, and a comprehensive workup for an infectious process and adequate antimicrobial coverage should always be undertaken. An analysis of a single-center cohort of patients receiving CART cell therapy (n=54, n=22 with Grade 3-4 CRS, n=16 included for analysis) and sepsis (n=108, n=80 eligible for analysis), identified 24 cytokines that discriminated between CRS and sepsis. It proposed a classification model in which IFNγ elevations >83pg/ml or low/moderate IFNγ-elevations <83pg/ml in conjunction with low IL1β <8pg/mml were associated with CRS whereas low IFNγ and IL1β >8pg/mml were consistent with sepsis with 97% accuracy (34).

Given the importance of biologic samples and biomarkers in understanding the pathophysiology of efficacy, toxicity and clinical management in the context of an ever-expanding field of novel products, efforts are under way to harmonize research efforts and provide a framework for biomarker discovery (35). For commercial products, most clinical centers voluntarily report toxicity and effectiveness data to the Center for International Blood and Marrow Transplant Research Cellular Immunotherapy Data Resource (CIDR) in a coordinated effort to capture outcomes (36). Likewise, a blueprint has been proposed for a collaborative effort between academia, industry, health authorities and professional societies. Clinical data reporting would be complemented by standardized collection and analysis of product, blood, marrow, tumor, and stool samples to produce comparable data on response and toxicity. This will require sharing of data and tools to allow for real time quantification and pharmacokinetics of CART cells post-infusion. Deposition of analysis results into a digital database would allow for comprehensive analysis and comparison across different centers and products and further the development of CLIA-approved assays that would support optimal clinical decision making for individual patients.

### CRS in Patients Receiving Bispecific Antibodies

While CRS is most recognized as an entity related to immune effector cells, it also emerged as an immunopharmacological response to bispecific T cell engaging antibodies (BiTEs) such as Blinatumomab (37). Blinatumomab consists of two single chain variable fragments, connected by a flexible linker, binding CD3 and CD19 respectively. This allows T cells to bind to the CD19 antigen on leukemia blasts and B-cells and leads to non-major histocompatibility-complex restricted T cell activation, activation of polyclonal T cells and killing of leukemia blasts and CD19+ B cells. It is currently FDA-approved for the treatment of adult and pediatric B-ALL in first or second complete remission with MRD ≥ 0.1% (38). In several trials investigating the use of Blinatumomab in adults, severe CRS occurred in 2-6% of patients, with neurologic toxicities in 13-17% of patients (39–41).

Blinatumomab is now frequently used in the therapy of relapsed/refractory pediatric B-ALL. In a single center retrospective analysis of 38 pediatric patients receiving Blinatumomab, febrile reactions during Blinatumomab infusion were frequent and occurred in 84% of patients. 53% of patients experienced CRS, 18% Grade 3 or 4 during the first cycle. Neurotoxicity was observed in 18% of patients (42). CRS and neurotoxicity typically occur in the first week of the first cycle of Blinatumomab, are less frequent with subsequent Blinatumomab cycles and can be mitigated by interruption of therapy, subsequent dose-reduction and if necessary corticosteroids and/or Tocilizumab administration (43).

During the Phase I/II prospective multicenter study of Blinatumomab in relapsed/refractory pediatric ALL, 3 of 5 DLTs in the dose finding phase were Grade 4 CRS events, one of which was successfully treated with Tocilizumab (44). A syndrome of CRS with features of HLH/MAS was highlighted in a separate case report by Teachey et al. (45). The patient developed high fevers, hyponatremia, hypotension, hypoxemic respiratory failure, accompanied by hyperferritinemia (22,000ng/mL), IL6 elevation (680pg/mL), cytopenias and hypofibrinogenemia. Other pertinent elevations in laboratory parameters included IL2R (4800pg/ml), IL10 (5338pg/mL), IFNγ (190pg/mL), MCP1 (2000pg/mL range), IL8 (400pg/ml range) and MIP1b. Tocilizumab was administered after continued clinical deterioration, despite discontinuation of Blinatumomab and was followed by dramatic improvement in both clinical and laboratory aberrations. In the trial, severe toxicity was subsequently mitigated by the administration of a stepwise Blinatumomab dosing approach (5ug/m^2^/d for the first 7 days, then 15ug/m^2^/d). In the cohort treated in this manner, 8 of 70 patients (11%) had CRS of any grade, while grade 3 or 4 CRS was observed in 6% of patients. Seventeen had neurologic/psychiatric events (24%) with seizures in two patients (3%) (44).

In a recent multicenter study investigating the use of Blinatumomab as post-reinduction consolidation therapy utilizing Dexamethasone premedication at the start of each cycle of continuous Blinatumomab infusion, CRS of any grade occurred in 22% of patients (Grade ≥3 in 1%), encephalopathy in 15% (Grade ≥ 3 in 4%) and seizure in 5% (Grade ≥ 3 in 1%), with the majority of events occurring during Cycle 1. Management of adverse events depended on severity and included treatment interruption, a short course of Dexamethasone (4 days followed by a rapid taper) and best supportive care. For patients experiencing toxicity, Blinatumomab could be resumed with dose reduction and Dexamethasone premedication once CRS/neurotoxicity resolved to ≤ Grade 1; a second AE requiring interruption of drug mandated permanent discontinuation (46).

### CRS in Patients Undergoing Haploidentical HCT

CRS is also increasingly well described in the setting of allogeneic HCT, specifically haploidentical HCT utilizing T-cell replete peripheral blood stem cell grafts. Historically, ex vivo T cell depletion was required for Haplo-HCT with methods such as CD34+ positive selection, CD3+ depletion or αβTCR-depletion. This resulted in very low doses of mismatched αβT cells in the graft. However, with the advent of Haplo-HCT using lymphocyte replete grafts and post-transplant Cyclophosphamide (PTCy), highly mismatched T-cells are infused in the graft and proliferate in response to alloantigens (47). This reaction proceeds unencumbered until PTCy is administered early after transplantation, typically on Day +3 and day +4. Cyclophosphamide selectively eliminates alloreactive T cells while sparing hematopoietic stem cells. High fever early after infusion of haploidentical grafts has been well described (48, 49), typically occurring in the first 3 days following graft infusion and subsiding after the second dose of Cyclophosphamide. More recently, the full syndrome of CRS has been described in several retrospective series of adult patients with severe CRS being most prominent in T-cell replete peripheral blood (PB) Haplo-HCT. This not surprising, given that G-CSF-mobilized peripheral stem cell grafts contain approximately 1 log-higher doses of T-cells than unmobilized bone marrow grafts. In a series of 146 consecutive adult patients (range 27-78 years) undergoing Haplo-HCT with PBSC between 2013-2017 at a single institution, CRS as graded by the Lee criteria (3) occurred in 89% of patients. Most cases were of mild severity, however severe (Grade 3-5) occurred in 17% of patients. Patients with severe CRS had a significantly higher NRM at 6 months than those with Grade 0-2 CRS (36% vs 8%, p<0.001) and lower 2-year OS (61% vs 40%) as well as a significant delay in neutrophil and platelet engraftment. Risk factors for severe CRS included recipient age >60years, and receipt of radiotherapy, but CD34+ or CD3+ cell doses were not identified as risk factors. A DRB1 mismatch in the graft-versus host direction appeared to be a requirement for the development of severe CRS (50).

Similar findings were also reported in a series of 107 consecutive Haplo-HCT patients receiving PTCy with >90% of patients having PBSC grafts. This series also evaluated 39 patients receiving HLA-identical HCT with PTCy as GVHD prophylaxis and found that CRS as defined by the original Lee criteria (3) occurred in 76% of Haplo-HCT patients compared to 14% of those receiving HLA-matched HCT with PTCy. CRS was typically grade 1 or 2 and was associated with the use of PBSC and a higher Total Nucleated Cell (TNC) dose. CRS rates were 83% in patients with TNC > 6x10^8^/kg compared to 53% in those who received lower TNC doses. CRS was associated with higher rates of grade II-IV aGVHD (60% vs 28.6%), but not with Grade III-IV aGVHD, cGVHD, NRM, EFS or OS (51).

In a different single-center cohort of 75 patients ranging from 19-73 years in age, CRS occurred in 87% of patients and severe treatment-related mortality in 12% of patients. Patients with severe CRS had higher treatment-related mortality as well as delayed neutrophil engraftment. In this cohort, cytokine levels were prospectively assessed in a subset of patients and high IL6 levels were found in 10 patients, 7 of whom received Tocilizumab. The median day of Tocilizumab treatment was d +3 (range 1-5), after which symptoms of CRS resolved within 48hrs and CRP levels dropped to below 50% of peak value. With a median follow-up of 196 days (range, 74-421 days), Tocilizumab administration did not appear to impact engraftment or efficacy of PTCy, although 1 patient subsequently developed steroid-refractory Grade IV acute liver GVHD (52). Importantly, none of the patients with severe CRS were noted to have a skin rash which may be clinically relevant for the distinction of CRS from engraftment syndrome or hyperacute GVHD (53).

Finally, a multicenter study by Abboud and colleagues retrospectively analyzed a cohort of 451 patients undergoing PTCy-based Haplo-HCT. This study confirmed a high overall CRS incidence of 90%, with the majority of cases mild (73%) and severe CRS occurring in 17%. Risk factors for severe CRS included the use of PBSC grafts, recipient CMV seropositivity, history of prior HCT and additionally HCT-CI and donor-recipient sex mismatch (in patients receiving PBSC grafts). Severe CRS was associated with a significant delay in neutrophil and platelet engraftment, but no differences in aGVHD and in fact lower rates of chronic GVHD. Interestingly the risk of relapse was significantly lower with both mild and severe CRS. However, with higher rates of NRM in the severe CRS group, OS was superior in patients with mild CRS. Management of severe CRS was center-dependent but included the use of steroids with or without Tocilizumab (54).

Further study is required to characterize long-term outcomes in those patients experiencing severe CRS and the impact and optimal timing of Tocilizumab therapy. Most of the literature describes adult patients, and CRS in the context of pediatric haplo-HCT is not well characterized. Differences would be expected from the adult experience at least partly because marrow is the most frequently used pediatric graft source and because severity of CRS appears to correlate with older age. However, severe CRS has been observed in pediatric Haplo-HCT (unpublished institutional data).

The incidence and management of CRS and associated toxicities in pediatric patients are likely to become further elucidated over the next years given the many advantages to the use of PTCy. This approach to HCT greatly expands the donor pool by allowing the use of haploidentical family donors. *In vivo* T cell depletion avoids the expense and sophisticated infrastructure required for ex vivo graft manipulation and can be offered outside of a research trial. As a result, PTCy-based haploidentical transplants have become increasingly used worldwide including in low and middle-resourced countries where access to alternative donors is limited. Tocilizumab is rarely available in these less resourced settings, due to expense and limited availability, particularly since its identification as a tool in the treatment of severe COVID infections. The approach to CRS has not been standardized in this context but high dose steroid administration at the first signs of CRS has been utilized. An alternative approach in the absence of tocilizumab, employs the use of lower dose steroids (0.5 mg/kg/day) as prophylaxis from day -1 before graft infusion through the administration of post-HCT Cyclophosphamide. This may be of particular importance when PBSC grafts are used in smaller patients who will receive a disproportionally high T cell dose. An alternative or complementary approach is to cap the CD34+ cell dose at 6-7.5x10^6^ CD34+ cells/kg/recipient body weight (Personal communication, Dr Elhaddad). These interventions appear to abrogate most cases of severe CRS and allow the use of haploidentical donors in the absence of access to targeted anti-cytokine therapies. There is theoretical concern that this early administration of steroids could blunt the expansion of alloreactive T cells that underlies the efficacy of GVHD protection with PTCy. Robust data reporting on outcomes including the incidence of GVHD and relapse will be required to fully assess the efficacy of this approach.

## Principles of Toxicity Management

### CRS Management

General management recommendations of IEC-related toxicities have been put forth by a number of working groups (3, 7, 11), but specific considerations apply to pediatric patients receiving Immune Effector Cells (55, 56). With rapid advances in the field, these recommendations will likely require modification over time to reflect toxicities emerging with novel agents, advances in our understanding of the pathophysiology and the availability of novel therapeutics to ameliorate toxicity while preserving efficacy. Key features of successful IEC toxicity management are close monitoring and early recognition of symptoms, provider and family education and an appropriate institutional infrastructure with involvement of the primary care team, pediatric intensive care unit, emergency room and pharmacy to facilitate assessment, workup, and management without delay (**Table 3**).

**Table 3:** CRS grading and management.

CRS Parameter	Grade 1	Grade 2	Grade 3	Grade 4
**Fever**	Temperature ≥ 38°C	Temperature ≥ 38°C	Temperature ≥ 38°C	Temperature ≥ 38°C
**Hypotension**	None	Not requiring vasopressors	**Requiring a vasopressor** with or without vasopressin	**Requiring multiple vasopressors** (excluding vasopressin)
**Hypoxia**	None	Requiring low-flow (O_2_ ≤ 6L/minute) nasal cannula or blow-by oxygen delivery	**Requiring high-flow** (O_2_>6L/min) nasal cannula, facemask, nonrebreather mask or Venturi mask	Requiring positive pressure (e.g. CPAP, BIPAP, intubation and mechanical ventilation)
*Grade is determined by the more severe event among CRS parameters. Grading adapted from Lee DW (9), Biol Blood Marrow Transplant 2019				
**Management**	⇩	⇩	⇩	⇩
**Supportive Measures**	-Blood cultures, ID workup -Antibiotics -Antipyretics/Cooling measures -Maintain hydration	Grade 1 interventions, **plus:** -IV Fluid bolus (x1-2) as needed -Consider stress-dose hydrocortisone -Respiratory support/O2 as needed	Grade 2 interventions, **plus:** -Transfer to ICU -Vasopressors as needed -Escalate respiratory support as needed	Grade 3 interventions, **plus:** -Maintain ICU level care -Escalate respiratory support as needed
**Tocilizumab**	Consider Tocilizumab for prolonged high fever	Consider Tocilizumab (Toci) for prolonged Grade 2 CRS	**Administer Tocilizumab**	
<30kg: 12mg/kg			May repeat Q8hr	
≥30kg: 8mg/kg			(maximum 3-4 doses)	
**Corticosteroids** -Methylprednisolone IV: 1-2mg/kg div q6-12h **OR** -Dexamethasone IV: 0.5-1mg/kg (max 10mg) Q6h			Consider steroids with 2^nd^ dose of Toci if CRS refractory to 1^st^ Toci dose	
			**Administer steroids for CRS refractory to 2 doses of Toci**	
**Third-line agents** Anakinra, Siltuximab, ATG HD Methylprednisone, Safety switches				Consider third-line agents if no improvement after 2 doses of Tocilizumab + steroids. Consider alternative etiologies

In bold are headers, key CRS parameters and management interventions, and key criteria determining CRS Grade as well as key interventions.

At the first onset of fever following IEC therapy, patients should be promptly evaluated and, if outpatient, admitted to the hospital. Evolving sepsis and other infectious processes are important in the differential diagnosis and thus blood cultures should be obtained promptly, and empiric antimicrobial coverage initiated. Additionally, imaging and/or additional infectious workup should be undertaken depending on the patient’s clinical situation. The presence or development of hypotension should be assessed using age-specific physiologic values (57) and the patient’s personal baseline blood pressures and prior need for antihypertensive agents. Once hypotension is identified, prompt management should ensue including an urgent evaluation by the critical care team. Initial fluid resuscitation with 10-20ml/kg IV normal saline should be performed after assessment of patient size, vulnerability to fluid shifts and preexisting conditions. Care must be taken not to induce acute fluid overload and potential respiratory failure in patients with capillary leak. The optimal fluid for resuscitation in this setting has not been established and consideration may be given to use of albumin in patients with hypoalbuminemia and capillary leak (58, 59). Providers should be prepared to escalate management with additional agents and to not exceed two fluid boluses before initiating vasopressors to support adequate end-organ perfusion as clinically indicated. If underlying adrenal insufficiency is suspected, stress-dose hydrocortisone should be initiated. Concurrently, patients should be closely monitored for CRS-associated coagulopathy and hypofibrinogenemia managed with fibrinogen concentrate or cryoprecipitate (12).

Tocilizumab, a monoclonal antibody which blocks the IL6 receptor and therefore the IL6 pathway, has emerged as an essential therapeutic to intervene in the inflammatory pathway of CRS and has been critical in the success of CART cell therapy by enabling the successful management of life-threatening toxicities without long-term impact on CART cell efficacy (31). It was approved by the FDA for the management of CRS in 2018 (60). Availability of at least 2 doses of Tocilizumab for a given patient should be confirmed prior to CART cell infusion. Tocilizumab is administered IV based on weight (Weight <30kg: 12mg/kg; Weight ≥30kg: 8mg/kg with a maximum dose of 800mg). Tocilizumab administration was initially reserved for patients requiring high-dose or multiple vasopressors, or those requiring ≥40% FiO2 support for hypoxia as well as those with Grade 3 or higher CTCAEv4.0 organ toxicity. However, as the field has gained more experience, earlier administration of Tocilizumab has emerged as a strategy to preempt high-grade CRS and has resulted in a lower incidence of Grade 3-4 CRS. As such, Tocilizumab administration should be considered for ASTCT Grade 2 CRS, particularly if prolonged and should be administered rapidly in patients with ≥Grade 3 per ASCTC grading criteria (Temperature ≥38°C, requiring a vasopressor and/or High-Flow Nasal Canula (HFNC), facemask, nonrebreather mask or Venturi mask). Recent ASCO guidelines also recommend consideration of Tocilizumab for patients with Grade 1 CRS and persistent (>3 days) or refractory fever (10). While repeat administration every 8 hours for up to 4 total doses is possible, clinical improvement in patients responding to Tocilizumab is generally observed within hours of initial administration (61). In patients who appear refractory to Tocilizumab, administration of corticosteroids either concurrently with the second dose of Tocilizumab or within 12-18 hours after the initial Tocilizumab dose should be considered. The type of corticosteroid used differs based on institutional preference but may include Methylprednisolone 1-2mg/kg/d IV daily or Dexamethasone 0.5-1mg/kg/dose (maximum 10-20mg) every 6 hours. Upon improvement to Grade 1 CRS, a rapid steroid taper should be initiated. Although the impact of early Tocilizumab and corticosteroid administration on long-term anti-leukemic efficacy has not been prospectively studied, no apparent detrimental effects on MRD-negative complete remission rates, LFS, OS, CART cell expansion or persistence has been observed with an early intervention strategy. In this approach, Tocilizumab is used for persistent symptoms of mild CRS with a focus on persistent fever ≥39°C for 10 hours, early hypotension and mild hypoxia; 5-10mg of Dexamethasone Q6-12 hours is given for patients experiencing sustained/recurrent fevers, requiring vasopressor support or requiring increasing respiratory support (62). Similarly, a risk-adapted strategy of Tocilizumab administration based on B-ALL tumor burden at the time of CART infusion was able to reduce the rate of Grade 4 CRS (19). In this study, patients with tumor burden of ≥40% on marrow evaluation prior to infusion received a single dose of Tocilizumab after the development of high, persistent fevers, whereas patients with <40% blast burden received standard CRS management. The use of Anakinra early in the course of IEC toxicity is currently being investigated.

In patients who do not respond to these measures, third-line agents should be entertained, including Siltuximab (63), Anakinra (29, 64) and high-dose Methylprednisolone (1gram daily x 3days, followed by rapid taper) (55). In products containing an inducible safety switch such as inducible caspase-9 (iC9) (65) this should be triggered in the face of serious uncontrollable toxicity. Although clinical data is lacking, preclinical models have suggested that the tyrosine kinase inhibitor Dasatinib may be utilized to transiently ablate CAR signaling by interfering with the lymphocyte-specific protein tyrosine kinase (LCK) and inhibiting phosphorylation of the CD3ζ-chain contained in the CAR (66) (**Figure 2**).

### ICANS Management

The risk for neurological toxicities differs based on the type of IEC administered, baseline neurologic status and disease burden. It has been observed in patients receiving IEC and Blinatumomab but is not currently characterized as a complication seen after Haplo-HCT. In general, a risk-adjusted approach, particularly for preemptive measures is recommended (11). Some centers have adopted a baseline evaluation by neurology prior to IEC infusion for all patients and this should definitively be considered in patients at high risk for ICANS. Additionally, the potential benefit of a baseline brain MRI warrants consideration.

There is currently insufficient evidence to recommend anti-seizure prophylaxis in all patients undergoing IEC therapies, and patients can develop seizures despite leviracetam prophylaxis (20). However, seizure prophylaxis with Leviracetam 10mg/kg (maximum 500mg/dose) every 12 hours should be considered in patients deemed at high risk for ICANS, such as pediatric patients with active CNS disease, a history of seizures or neurologic abnormalities on imaging. Initiation of Leviracetam may also be considered in patients developing high grade CRS and/or early symptoms of ICANS. Although the ideal dose and duration have not been determined, Leviracetam is the anti-epileptic drug of choice both for the prophylaxis and treatment of seizures during IEC therapy, owing to its low risk of cardiac toxicity, safe use in patients with hepatic dysfunction and lack of impact on cytokine levels (7).

The recognition of ICANS, particularly in young children, requires a high degree of suspicion, close communication with parents/caregivers and regular screening during the period of highest risk. The ASTCT ICANS criteria should be used not only for grading but also screening purposes. In conjunction with assessment of the level of consciousness, presence or absence of seizure activity, motor weakness or elevated ICP/cerebral edema, the Immune Effector-Cell associated Encephalopathy (ICE) score should be utilized in children >12 years if developmentally appropriate (**Table 4**), and the Cornell Assessment of Paediatric Delirium (CAPD) scoring in children <12 years old and those who are developmentally unable to be evaluated using the ICE score (**Table 5**). A CAPD score >/=9 or a significant rise in CAPD score from baseline should raise concern for delirium. A baseline evaluation should be undertaken in all patients prior to infusion. Thereafter patients, should be regularly monitored. During the expected high-risk period, performing a formal ICANS assessment twice daily has been suggested (56).

**Table 4:** ICE Scoring.

ICE Scoring (Children ≥ 12 years)			
		Points:	Maximum points
**Orientation**	Year		1
	Month		1
	City		1
	Hospital		1
**Naming**	Object 1 (e.g. clock)		1
	Object 2 (e.g. pen)		1
	Object 3 (e.g. button)		1
**Following Commands**	Ability to follow a simple command (e.g. “Show me 2 fingers” or “Close your eyes and stick out your tongue”)		1
**Writing**	Ability to write a standard sentence (e.g. “Our national bird is the bald eagle”)		1
**Attention**	Ability to count backwards from 100 by 10		1
**Total Points:** (ICANS Grade 1: 7-9, Grade 2: 3-6, Grade 3: 0-2)			**10**

Adapted from Lee DW (9), Biol Blood Marrow Transplant 2019. In bold are Headers and Key domains of ICE scoring.

**Table 5:** CAPD Scoring.

CAPD Encephalopathy Assessment for Children < 12years						
Point assignment:	Never (4 points)	Rarely (3 points)	Sometimes (2 points)	Often (1 point)	Always (0 point)	Guidance for patients age 1-2years:
1. Does the child make eye contact with the caregiver?						Holds gaze, prefers parent, looks at speaker
2. Are the child’s actions purposeful?						Reaches and manipulates objectes, tries to change position, if mobile may try to get up
3. Is the child aware of his/her surroundings?						Prefers primary parent, upset when separated from pereferred caregivers. Comforted by familiar objects
4. Does the child communicate needs and wants?						Uses single words or signs
	**Never**	**Rarely**	**Sometimes**	**Often**	**Always**	
	**(0 points)**	**(1 point)**	**(2 points)**	**(3 points)**	**(4 points)**	
						
5. Is the child restless?						No sustained calm state
6. Is the child inconsolable?						Not soothed by usual comforting actions
7. Is the child underactive; very little movement while awake?						Little if any play, efforts to sit up, pullup, and if mobile crawl or walk around
8. Does it take the child a long time to respond to interactions?						Not following simple directions. If verbal, not engaging in simple dialoge withwords or jargon
**Total Points:** (ICANS Grade 1 and 2: 1-8, Grade 3: ≥ 9)						

Adapted from Traube et al. (67).

If signs and symptoms consistent with ICANS develop (**Table 6**), the frequency of neurologic monitoring should be increased, and a comprehensive diagnostic workup undertaken in consultation with a pediatric neurologist. Neuroimaging, diagnostic lumbar puncture and electroencephalography are often warranted to rule out alternative etiologies including infections (10).

**Table 6:** ICANS grading and management.

Neurotoxicity Domain	Grade 1		Grade 2	Grade 3		Grade 4
**≥12 years: ICE score OR**	7-9		3-6	0-2		0 (unarousable and unable to perform ICE)
**<12 years: CAPD score**	1-8		1-8	≥9		Unable to perform CAPD
**Depressed level of consciousness (any age)**	Awakens spontaneously		Awakens to voice	Awakens only to tactile stimulus		Unarousable or requires vigorous or repetitive tactile stimuli to arouse; stupor coma
**Seizure (any age)**	None		None	Any clinical seizure (focal or generalized) that resolves rapidly OR nonconvulsive seizures on EEG that resolve with intervention		Life-threatening prolonged seizure (.5min); or repetitive clinical or electrical seizures without return to baseline in between
**Motor weakness (any age)**	None		None	None		Deep focal motor weakness, such as hemiparesis or paraparesis
**Elevated ICP/cerebral edema**	None		None	Focal/local edema on neuroimaging		Decerebrate or decorticate posturing, cranial nerve VI palsy, papilledema, Cushing’s triad or signs of diffuse cerebral edema on imaging
*Grade is determined by the more severe event among neurotoxicity domains not attributable to any other cause. Grading adapted from Lee DW (9), Biol Blood Marrow Transplant 2019						
**Management**	**Management should be tailored to the type of CART product and patient characteristics**						
**Supportive Measures**	-Close monitoring with ICE/CAPD scoring ≤ q6hrs -Initiate workup as indicated -Airway protection -Aspiration precautions -Convert medications, nutrition to IV as indicated -Avoid pharmacologic CNS depression	Grade 1, **plus**: -EEG -Neuroimaging -initiate further workup as indicated			Grade 2, **plus**: -ICU transfer -LP -consider repeat neuroimaging q2-3 days	Grade 3, **plus**: -ICU transfer -Neuroprotective measures: Head of bed to 30° and neck midline, normothermia, normocarbia (paCO2 35-40mmHg), euglycemia, eunatremia -Increased ICP/Herniation: Initiate hyperosmolar therapy, deep sedation, hyperventilation (paCO2 30-35mmHg), stat head CT, neurosurgery consultation	
**Work-up:**							
-Neurology consultation -Neuroimaging (MRI with and without contrast or non-contrast Head CT; MRI spine if focal symptoms) -Fundoscopy -Electroencephalography (EEG) -Lumbar puncture (LP)							
**Antiseizure Medications**	Consider Leviracetam prophylaxis in high-risk patients				Treat seizures with Leviracetam/Benzodiazepines	Treat seizures with Leviracetam/ Benzodiazepines	
**Corticosteroids**		Consider Steroids. >For concurrent low- grade only CRS, prioritize steroids over Tocilizumab			Initiate Steroids (at least 2 doses)	Initiate Steroids (at least 2 doses)		
-Methylprednisolone IV: 1-2mg/kg div q6-12h **OR** -Dexamethasone IV: 1mg/kg (max 20mg) Q6h						For Grade 4 or focal edema consider: Methylprednisolone IV 30mg/kg/daily (Max 1gram/d)	

In bold are Headers, Key ICANS criteria and interventions used in management.

Neuroimaging by Brain Magnetic Resonance Imaging (MRI) is preferred but if this is not feasible based on the patient’s stability or challenges around required sedation, Computer Tomography (CT) imaging may be obtained. Radiographic findings may vary significantly among patients with ICANS and range from normal (20) to severe findings of intracranial hemorrhage, infarcts, and diffuse edema (15). However, common patterns include reversible T2 hyperintensities and swelling in the bilateral thalami, pons, and medulla (in a pattern similar to the rare central variant of posterior reversible encephalopathy syndrome), frequently accompanied by symmetric white matter T2 hyperintensities that are subcortical or affect the external and extreme capsule. Focal white matter T2 hyperintensities with or without contrast enhancement may occur at sites of prior CNS injury. Furthermore, cortical diffusion restriction with subsequent cortical atrophy is a rare variant (20, 68, 69).

EEG in pediatric patients with neurotoxicity may reveal diffuse background slowing indicative of diffuse encephalopathy and may capture additional seizures in some patients (69). The most common EEG finding in adults was frontal intermittent rhythmic delta activity (FIRDA) and diffuse or frontal slowing with or without triphasic waves (2-3Hz). Nonconvulsive status epilepticus can also been observed (7, 20).

Characteristic CSF findings in patients with ICANS may include pleocytosis relative to peripheral WBC counts, high CSF protein levels and serum CSF/serum Albumin quotients (Qalb). CART cells can be detected in the CSF by product-specific qPCR assays (20) although results are often not able to be clinically used. Preclinical models suggest migration of CART as well as non-transduced lymphocytes into the CSF (30) and the quantity of CART cells in the CSF does not appear to correlate with neurotoxicity severity (20).

A higher incidence of Disseminated Intravascular Coagulation (DIC) has been reported in patients with severe neurotoxicity and coagulation parameters should be closely monitored and corrected. High grade CRS is associated with the development of neurotoxicity and initiation of Leviracetam prophylaxis may be considered in those patients. High grade CRS, with or without co-occurrence of ICANS should be treated with Tocilizumab as outlined above. However, Tocilizumab does not penetrate the CNS and has failed to resolve symptoms of ICANS despite alleviating severe CRS. It has been postulated that Tocilizumab may in fact worsen neurotoxicity by at least transiently increasing IL6 concentrations in the CSF (21, 63, 70). Therefore, the management of neurotoxicity with steroids may take precedence over the management of low-grade CRS (11). As an example, Grade ≥2 ICANS with Grade 1 CRS should be preferentially managed with steroids. However, for high-grade CRS in conjunction with ICANS, Tocilizumab should be used in conjunction with steroids. The choice of corticosteroid differs among institutions and there are product-specific recommendations but in general Dexamethasone 1mg/kg (max 20mg) every 6 hours or Methylprednisolone IV 1-2mg/kg/day divided Q6-12hours are used. For Grade 4 ICANS or cerebral edema, high grade Methylprednisolone (30mg/kg/day, Max 1gram/d) should be utilized. Seizures should be managed per institutional standard. Benzodiazepine therapy is typically used to abort seizures, while Leviracetam is used as the antiepileptic medication of choice given its low risk of cardiotoxicity, safe use in patients with hepatic dysfunction and the fact that it does not affect cytokine levels (7, 11, 55). Transfer to a pediatric ICU should be undertaken for grade ≥3 ICANS, progressive ICANS or ICANS unresponsive to therapy. Patients should be closely monitored for the development of cerebral edema. In patients with evidence of increased intracranial pressure (papilledema, elevated CSF opening pressure ≥20mmgHg, or cerebral edema on neuroimaging), intensive treatment algorithms per institutional standard should be rapidly employed which may include hyperventilation strategies, hyperosmolar therapy, elevation of the head of bed and consultation with neurosurgery (**Table 6**) (55).

### CarHLH Management

The clinical features observed in CRS overlap substantially with CarHLH (33). Therefore, standard CRS management (tocilizumab, corticosteroids) is indicated when symptoms of HLH are concurrent with CRS. However, with the investigation of novel CART-cell constructs, such as CD22-CAR T cells, a secondary inflammatory phase mimicking HLH has been observed and the term CarHLH suggested to aid in the distinction from CRS. While symptoms can overlap with severe CRS, this entity may occur after resolution of clinical symptoms consistent with CRS such as fever and hypotension. Prominent features of CarHLH include delayed coagulopathy characterized by disproportionally severe hypofibrinogenemia compared to PT/PTT abnormalities, significant hyperferritinemia, hepatic dysfunction and cytopenias (22) (**Table 7**). Based on current ASCO guidelines, a full workup to additionally include serum triglycerides, soluble IL-2 receptor alpha (sCD25 or sIL-2R) and/or CXCL9, bone marrow evaluation, lumbar puncture with CSF analysis and brain MRI with and without contrast workup for infectious triggers may be considered (10). Late-onset tocilizumab-refractory HLH-like symptoms may therefore represent a distinct pathology requiring tailored treatment approaches. IL1β-levels are high in patients with CarHLH and support the use of Anakinra (22). In this setting, Anakinra alone or in conjunction with corticosteroids has been used successfully to resolve HLH/MAS-like toxicities without any apparent negative effects on CAR T cell expansion or response (18). Additionally, coagulopathy should be closely monitored and managed by repletion of fibrinogen with cryoprecipitate. Current SITC guidelines therefore recommend consideration of third-line CRS agents such as Anakinra and corticosteroid for Tocilizumab-refractory late-onset HLH/MAS-like pathology. Etoposide should only be administered as a last resort (11, 71). Further investigations into this evolving area are likely to more clearly elucidate the clinical entity of CarHLH, the impact of early use of Anakinra as well the utilization of other therapeutic agents. High levels of IFNγ have been observed in patients with CarHLH, providing rationale for the potential use of Emapalumab, an anti-IFNγ monoclonal antibody (72), which is FDA approved for the treatment of primary HLH. However, clinical experience with Emapalumab in CarHLH is currently lacking and preclinical data raises concern regarding its possible negative impact on CART cell efficacy (73). Implications of CarHLH include severe infections (27) and multiorgan dysfunction highlighting the importance of robust supportive care for this patient population.

**Table 7:** CarHLH manifestations and management.

CarHLH Criteria			Car HLH manifestations	
Major Criteria (Both required)			Cytokine release syndrome (prior or concurrent)	
			Hyperferritinemia	
Minor Criteria			Hepatic transaminases ≥ Grade 3 or bilirubin ≥ Grade 3	
(At least ≥ 2 criteria)				
			Pulmonary manifestations ≥ Grade 3 (such as edema or hypoxia)	
			Renal insufficiency ≥ Grade 3	
			Coagulopathy	
			Evidence of hemophagocytosis on bone marrow evaluation	
Other supporting laboratory manifestations			Hypertriglyceridemia Cytopenias	

**Management**	**CarHLH concurrent with CRS**	**CarHLH after CRS or unresponsive to Toci**		**CarHLH unresponsive to CRS management and Anakinra**
**Supportive Measures**	Provide best supportive care per CRS management algorithm			
**Tocilizumab**	**Administer Tocilizumab based on CRS management algorithm**			
**Corticosteroids**	**Administer steroids based on CRS management algorithm**			
**Anakinra**		**Administer Anakinra**		
**Forth-line Agents**				**Consider administration of Etoposide**
-Etoposide				
(Consideration may be given to Emapalumab, Ruxolitinib)				The role of alternative agents is not yet established in CarHLH management

Adapted from Lichtenstein D (22), Blood 2021. In bold are headers and key management interventions.

### Critical Care Considerations for Toxicity Management

Up to 50% of pediatric patients who develop CRS will end up requiring pediatric intensive care unit (PICU) level care (16, 61). While the threshold for PICU transfer varies by institution, typically Grades 3 and 4 CRS require intensive care, while some Grade 2 CRS patients may be transferred to the ICU based on institutional protocol and clinical judgement of the patient’s trajectory. Among pediatric CAR-T patients, the most common complications requiring ICU-level care are hemodynamic instability (25-35%), respiratory insufficiency (15-25%), and neurologic instability (10-20%) (16, 61). It is worth considering that these numbers are likely to change both as institutions gain more experience with CAR-T therapy and its toxicities and as the eligible patient population is expanded.

Hemodynamic instability in CRS patients can be multifactorial and is thought to share common features with other hyperinflammatory states such as SIRS and sepsis. Inflammatory cytokines cause vasoplegia and capillary leak, promoting a distributive shock state. Additionally, inflammation can cause cardiac dysfunction and arrhythmias (74, 75). Acute hemodynamic stabilization of a patient with CRS should include rapid but judicious fluid administration to replete intravascular volume and protect end organ perfusion. In patients with persistent hypotension, there should be a low threshold for initiation of pressors and immediate PICU transfer. These patients should have an EKG, chest x-ray, and echocardiogram upon PICU admission. The choice of pressor follows similar reasoning to that for patients with septic shock. Most CRS patients present with a “warm shock” picture, and norepinephrine can be used to improve vascular tone while providing some inotropy and chronotropy. Epinephrine can be considered in patients with hemodynamically significant myocardial dysfunction. Additional vasoactives such as vasopressin, phenylephrine, and milrinone have been utilized as the patient’s physiology demands (74). In all cases, pressors should be titrated to normotension for age, and patients with pressor-refractory hypotension should receive stress-dose steroids. Acute presentation of CRS is indistinguishable from sepsis, and CAR-T patients are profoundly immunosuppressed and prone to serious bacterial infections. In one study, 15% of adult B-ALL patients developed bacteremia within 28 days of receiving CAR-T infusion (76). Therefore, any CAR-T patient with hemodynamic instability should be cultured and promptly started on broad-spectrum antibiotics. As with septic patients, fluid management in CRS necessitates a delicate balance between repleting intravascular volume and avoiding excessive capillary leak and third spacing. Central venous pressure can be used to assess volume status, and in patients who are clinically euvolemic, pressors should be prioritized to maintain blood pressure. In sicker patients, surrogates of organ perfusion and oxygen delivery/consumption such as lactate and mixed venous saturation may be trended to guide hemodynamic support.

Respiratory insufficiency is common in CRS and is thought to be primarily due to pulmonary edema from capillary leak (77). Additionally, myocardial dysfunction can cause cardiogenic pulmonary edema, and CRS patients can develop large pleural effusions that impair respiratory mechanics. CRS-associated respiratory insufficiency generally falls on the ARDS spectrum, and is typified by alveolar fluid accumulation, surfactant dysfunction, and poor lung compliance, leading to impaired oxygenation (9, 61). Patients with Grade 2 CRS respond to supplemental oxygen alone, while Grade 3 and 4 CRS require ICU-level respiratory support. Support should be titrated in a stepwise manner with the goal of alveolar recruitment and restoration of adequate gas exchange. Many patients will respond well to HFNC or Continuous Positive Airway Pressure (CPAP)/Bilevel Positive Airway Pressure (BiPAP). Patients with severely impaired respiratory mechanics and gas exchange may require intubation and mechanical ventilation. In these patients, an ARDS ventilation strategy is recommended with high PEEP and low tidal volumes (4-6 ml/kg). Arterial blood gas analysis should direct ventilator titration and weaning. Careful fluid management is critical, and fluid restriction and gentle diuresis should be considered as the patient’s end organ perfusion allows (78). Other interventions such as proning and nitric oxide can be useful in improving refractory hypoxemia, but data on outcome improvement in the pediatric population are mixed (79, 80). Finally, patients with large pleural fluid collections may require drainage and possibly chest tube placement to improve respiratory mechanics.

ICANS is a well-described CRS-adjacent neurotoxic entity whose pathophysiology is incompletely understood. It is thought to result from inflammatory cytokines causing cerebral endothelial dysfunction and blood-brain-barrier disruption with resulting neuroinflammation (21, 81). Clinically, mild (Grade 1-2) ICANS presents with encephalopathy and behavioral dysregulation (**Tables 4** **–** **6**). More severe (Grade 3-4) ICANS will require ICU-level care, and can involve seizures, cerebral edema, and rarely herniation (9, 81). Initial management of a patient with ICANS should include close serial neurologic examination and bedside fundoscopy. Evolution of encephalopathy can be trended by ICE score in children > 12 years old, or by CAPD score in younger children and in patients who are developmentally unable to be evaluated using the ICE score (**Tables 4**, **5**) (9, 82). Lumbar puncture can be helpful to evaluate for infection and inflammation and should be considered in conjunction with cross-sectional imaging if there is concern for cerebral edema or hemorrhage. EEG should be performed in any patient with concern for seizure, including sudden changes in mental status. Patients with concern for recurrent seizures or non-convulsive status epilepticus should have continuous EEG monitoring to help direct therapy. In patients with radiologic or clinical evidence of cerebral edema, neuroprotective measures should be implemented, and high-dose corticosteroids should be initiated (**Table 6**) (55). While many patients with ICANS will have already received Tocilizumab for CRS, Tocilizumab does not efficiently cross the blood-brain barrier, does not appear to abrogate neurotoxic symptoms (20, 83), and is therefore not recommended for primary management of ICANS. Patients with clinical signs of increased intracranial pressure (such as Cushing’s triad or evidence of herniation syndromes) should be ventilated to a target p_a_CO_2_ of 30-35 mmHg (p_a_CO_2_ as low as 25 mm Hg can be used to acutely stabilize a patient with impending herniation) and have hyperosmolar therapy initiated while pursuing urgent neuroimaging (84). These patients should have neurosurgical evaluation for invasive intracranial pressure monitoring and possible surgical decompression. Of note, non-invasive ICP monitoring modalities such as transcranial doppler (TCD) and optic nerve sheath diameter have been studied, but invasive techniques remain the gold standard due to their superior accuracy (85).

Numerous other organ toxicities can be seen in patients admitted to the PICU with severe CRS. These include renal dysfunction requiring renal replacement therapy, liver inflammation resulting in hepatic synthetic dysfunction and coagulopathy, and hematologic abnormalities including cytopenias from CRS-related HLH. While management of these complications is outside of the scope of this article, they should be managed according to established standards of critical care.

## Conclusion

The field of pediatric IEC and HCT from alternative donors has made dramatic clinical and scientific advances in the last decade and continues to evolve rapidly. The signs and symptoms, timeline, risk factors and mechanisms of toxicities as well as pathways for therapeutic intervention and current management recommendations summarized in this review reflect the current experience, which is largely focused on the use of T-cell based or T-cell-engaging therapies for hematologic malignancies. The exploration of novel targets, approaches to achieve efficacy of immune effector cell strategies for solid tumors, increased utilization of allogeneic immune effector cells and highly sophisticated genetic editing of immune cell products are likely to uncover novel toxicities and further elucidate the biologic pathways involved. Likewise, judicious clinical studies of preempting and/or mitigating novel and currently recognized toxicities based on our understanding of the involved mechanisms are poised to spare this vulnerable patient population toxicities without compromising efficacy of these powerful therapies.

## Author Contributions

SB and GM wrote the manuscript. AE and LL contributed key portions, reviewed, and edited the manuscript. All authors contributed to the article and approved the submitted version.

## Conflict of Interest

The authors declare that the research was conducted in the absence of any commercial or financial relationships that could be construed as a potential conflict of interest.

## Publisher’s Note

All claims expressed in this article are solely those of the authors and do not necessarily represent those of their affiliated organizations, or those of the publisher, the editors and the reviewers. Any product that may be evaluated in this article, or claim that may be made by its manufacturer, is not guaranteed or endorsed by the publisher.
